# Racism in European Health Care: Structural Violence and Beyond

**DOI:** 10.1177/1049732320931430

**Published:** 2020-06-16

**Authors:** Sarah Hamed, Suruchi Thapar-Björkert, Hannah Bradby, Beth Maina Ahlberg

**Affiliations:** 1 Uppsala University, Uppsala, Sweden; 2 Department of Government, Uppsala University, Uppsala, Sweden; 3 Department of Sociology, Uppsala University, Uppsala, Sweden

**Keywords:** racism, structural violence, health care, qualitative research, Europe

## Abstract

Research shows how racism can negatively affect access to health care and treatment. However, limited theoretical research exists on conceptualizing racism in health care. In this article, we use structural violence as a theoretical tool to understand how racism as an institutionalized social structure is enacted in subtle ways and how the “violence” built into forms of social organization is rendered invisible through repetition and routinization. We draw on interviews with health care users from three European countries, namely, Sweden, Germany, and Portugal to demonstrate how two interrelated processes of unequal access to resources and inequalities in power can lead to the silencing of suffering and erosion of dignity, respectively. The strength of this article lies in illuminating the mechanisms of subtle racism that damages individuals and leads to loss of trust in health care. It is imperative to address these issues to ensure a responsive and equal health care for all users.

## Introduction

The effect of racism on health notwithstanding (Williams & Mohammed, 2013), limited theoretical exploration has looked at how racism is conceptualized in health care, specifically the subtle and invisible hard-to-detect workings of racism in health care encounters (Bradby, 2010; Bradby et al., 2019). According to Nazroo et al. (2019), current research on racism in health care lacks a theoretical focus on processes which lead to the (re)production of racial inequalities in health care and how these shape health care encounters. In this article, our contribution lies in theoretically framing racism in health care through the lens of structural violence. Our rationale for choosing structural violence is as follows. Structural violence enables us to (a) understand how racism as an institutionalized social structure is enacted through human practice in subtle, invisible ways; (b) analyze how structures and stratifications of inequality which underpin racism are normalized, naturalized, and taken for granted; (c) analyze the processes through which structures which systematically disadvantage racialized subject can coexist with care; and finally (d) allow us to see the “violence” built into given forms of social organization which is rendered invisible through its reutilization.

Our article draws on the experiential accounts of service users to understand racism in health care in three European countries. The main objective is to explore access to health care in several neighborhoods by interviewing local health care users. Our article is delineated as follows. First, we provide a brief overview of racism in health care and of challenges to conceptualizing racism in health care. Second, we exemplify the working of racism as structural violence through two processes with tangible outcomes. Third, we provide an overview of our qualitative methodology where we use individual in-depth interview narratives to analyze the social and economic processes that nurture inequalities. In the final section, we offer some concluding thoughts.

### Race, Racism, and Health Care

With the abandonment of the biological notion of “race” (see Goldberg, 2006; Miles, 1989), race came to be regarded as a questionable analytic social category. ln his analysis of the legislation against racism in Europe, Möschel (2011) argues that “race” is silenced in normative and legal discourse as well as in the implementation of the few legal instruments designed to protect against racism. This contributes to what Goldberg (2006) refers to as “racial evaporization” which reinforces the idea that racism belongs to the past, as well as what Bonilla-Silva (2009) refers to as racism without racists, that is, a new form of racism that is less overt and more difficult to pinpoint. Racism thus becomes an individual aberration (Bradby et al., 2019) and not a structural societal issue (Titley, 2016). How do we acknowledge minorities’ undeniable experiences of racism as real, when racism is no longer seen as a problem in modern welfare states or as (Appiah, 1996, p.81) states “the label works despite the absence of an essence”?

An established body of literature shows how racism increases the risk of morbidity (such as higher risk of hypertension, higher risk for poor mental health outcomes, and even low birth weight of infants) and mortality of ethnic minorities (Krieger, 2014). Experiences of racism have been shown to have negative effects on the physical and mental health of African Americans (Jackson et al., 1996). Both individual and institutional measures of perceived racism predict lower levels of mental health of Chinese Americans, even after controlling for various variables, including health insurance, socioeconomic variables, sex, age, and education (Gee, 2002). Racialization and class were found to have independent negative health consequences for ethnic minority people living in Britain (Karlsen & Nazroo, 2002a). Not only is the experience of racism associated with negative health status, but fear of racism has a negative impact on health outcomes, independent of the effect of gender, age, and household social class (Karlsen & Nazroo, 2004).

Racism operates through complex mechanisms, compromising social support which has an effect on health status and access to health care (Karlsen & Nazroo, 2002b). Institutional racism can also lead to the identification and reification of ethnic minority groups as different (Miles, 1989), as well as their social and economic exclusion (Paradies et al., 2015). In health care, racism has been shown to be associated with health service use outcomes such as lack of adherence to treatment uptake and delay in receiving adequate health care (Ben et al., 2017). In Sweden, for example, foreign-born patients have been shown to be less likely to receive adequate care in regard to heart failure medications as well as receiving lower rates of beta blockers than medically recommended (National Board of Health and Welfare, 2008). Asian and Black patients were found to be less likely to receive kidney transplant compared with White patients in a study in 36 European countries (Tjaden et al., 2016). Perceived racism is associated with lack of trust in health care and with refraining from seeking health care (Wamala et al., 2007). African Americans have been shown to receive lower quality pain control, even when having health care insurance, suggesting that their pain is not taken seriously (Green et al., 2003). Health care users experiencing racial discrimination also report being left out of decision-making processes concerning their health care (Peek et al., 2010). Implicit racial bias and unequal care provision have been reported by health care users from various health care providers (Thomas, 2018). As a way to understand the conundrum of racism, its invisibility, and obfuscation, on one hand, and its material consequences that shape the risk of morbidity and mortality, on the other, we frame racism through the theoretical lens of structural violence.

### Structural Violence

The concept of structural violence has been used to explain wide-ranging phenomena, including the effects of development agencies in Rwanda (Uvin, 1998), the culturally inflicted psychological suffering of bachelor farmers in Ireland (Scheper-Hughes, 1979), post–Cold War lessons from El Salvador (Bourgois, 2001), and the roots of the Israeli–Palestinian conflict (Rotberg, 2006). Coined by Johan Galtung (1969), structural violence is an invisible, indirect, and insidious process, and is often embedded in long-standing “ubiquitous social structures” that are normalized by stable institutions (Gilligan, 1997). According to Price (2012), “[in] order to see violence, one must see the structures” (p. 6). Similarly, Dilts et al. (2012) argue that rather than limiting ourselves to “agents and intentions” it is violence “built into structures, institutions, ideologies, and histories” that is important (p. 191). Farmer (2003) refers to structural violence as including a host of offenses against human dignity: poverty, social inequalities ranging from racism to gender inequality, and human rights abuses—with some abuses arising as a punishment for escaping structural violence (p. 8). This is what he refers to as the “multi-axial models of suffering,” that is, how individual biographies, embedded in the larger matrix of culture, history, and political economy, come to be translated as personal distress, suffering, and disease (Farmer, 1996, p. 272). On a similar track, Kleinman and colleagues (1997) argue that social suffering results “from what political, economic and institutional power does to people, and, reciprocally, from how these forms of power themselves influence responses to social problems” (p. ix). Medical anthropologists such as Das, Farmer, and Scheper-Hughes argue in relation to their research in India, Haiti, and Brazil that social forces and processes come to be embodied as biological events and create differing legacies of health and disease and outcomes thereof. As Farmer and colleagues (2006) state,

[the impact of ] large-scale social forces—racism, gender inequality, poverty, political science and war, and sometimes the very policies that address them—often determine who falls ill and who has access to care. (p. 1)

In fact a “simultaneous” consideration of these intersectional vectors of oppression enables us to understand “a political economy of brutality,” rather than the focus of any single axis to define the increased risk for extreme human suffering (Farmer, 1996, p. 274). In advanced industrialized societies and in modern bureaucratic and welfare states, Nancy Scheper-Hughes (1992) argues that a whole array of educational, social welfare, medical, psychiatric, and legal experts collaborate in the management and control of sentiments and practices that threaten the stability of the state and the fragile consensus on which its claim to legitimacy is based (p. 221). She defines this “everyday violence” as the “little routines and enactments of violence” practiced normatively in various administrative and bureaucratic settings such as in families, the educational system, schools, hospitals, and medical clinics (Scheper-Hughes, 1995, p. 143; 2004). This everyday violence is rendered invisible as it is “perceived” as the status quo, but “experienced” (see Price, 2012) as assaults on dignity and integrity, leading to a steady constraint or erosion of an individual’s agency. This invisibility is not because it is hidden but precisely the opposite—it is hardest to perceive because it is right before our eyes. Arguably, it is through repetition and reiteration that violence becomes invisible and assumed to be a normal status quo.

Structural violence operates at different though intersecting systemic levels (local, national, and global), and is built into structures (reproduced by social actors themselves) (see Giddens, 2013) of cultural and social institutions and shows up in unequal power, uneven distribution of resources, and consequently in unequal life choices and chances (also see Kohler & Alcock, 1976). Furthermore, structural inequalities (e.g., racial inequality) and structures of domination are “the product of an incessant labour of reproduction” (Pierre, 2004, p. 339), naturalized through repeated practice and so assumed to be part of the natural order of things. We do not refer here to acts of violence perpetrated by any identifiable subjects but rather to the “social machinery of oppression” (Farmer, 2004, p. 307). This, we argue, inadvertently leads to (a) the marginalization of specific groups and populations thus constraining their agency, (b) thereby sustaining inequalities through unequal access to power and resources and leading to (c) unavoidable illness, injury, and untimely death.

As institutions can nurture environments where medical staff view certain practices and relationships as part of a normal medical routine and with time, these practices (which can be perceived as disempowering by patients) become embedded in the culture of the clinical setting and routine. As Galtung (1969) in his seminal essay pointed out, if a person dies “despite all the medical resources in the world,” then structural violence is present (Galtung, 1969, p. 168). Furthermore, social processes turn information into meaningful knowledge and knowledge into action. Knowledge that is generated and validated through social processes becomes embedded in taken-for-granted assumptions and practice. Practice becomes institutionalized and no longer examined, evaluated, or criticized. The perceived stability and normalcy of the clinical setting masks a deeper and more pervasive violence which casts sufferers out of the public’s moral community to “muffle” suffering, rendering it barely noticeable (Morris, 1997; Opotow, 2000).

Several scholars have used the concept of structural violence to understand how socioeconomic and political forces shape the “landscape of risk” (Kelly, 2005, p. 721) with respect to morbidity and mortality experiences and also the contexts in which health care is provided, such as hospitals and clinics: how the health of female sex workers in Kathmandu is damaged (Basnyat, 2017), the disproportionate HIV transmission among African American men (Lane et al., 2004), the impact of living in disadvantaged communities on youth smokers (Lewis & Russell, 2013) as well as living with schizophrenia (Kelly, 2005). Lane et al. (2008) show how the public health approach for solving health disparities relies on “having each person take responsibility for his or her own health” (Lane et al., 2008, p. 417). Hole et al. (2015), in relation to health care services for aboriginal people in Okanagan Valley (British Columbia, Canada), suggest that the “production” of structural violence occurs through notions of visibility and invisibility, the conflation of equality with equity, and the disregard of Aboriginal values and beliefs (p. 1670). This extant body of literature has highlighted the usefulness of structural violence as a theoretical tool to understand how adverse health outcomes occur. Nonetheless, the processes of structural violence that yield negative health outcomes remain empirically underexplored.

In this article, we use the empirically underexplored concept of structural violence to theoretically frame racism in health care. Our use of the concept of structural violence allows the reader to consider the impact of subtle racism on patient experiences of health care services and how racism maintains and increases health inequities. We analyze the narratives of health care users in three European countries in regard to their experience of racism in health care and unveil the processes within health care through which structural violence is nurtured and reproduced. Consistent with the discourse on structural violence, we delineate two processes that illustrate the workings of structural violence in health care: (a) unequal access to resources that leads to the silencing of suffering and (b) inequalities in power, which lead to the erosion of dignity.

## Method

### Participant Recruitment

This study was part of a larger project, which aimed to examine health care users’ experiences of accessing and communicating with health care providers in eight neighborhoods in four European countries, namely, Sweden, Portugal, Germany, and the United Kingdom (Phillimore et al., 2015). Prior to the recruitment of residents, ethnographic observations were employed by researchers in the neighborhoods to identify different health care resources and engage in conversations with residents and health care providers. The ethnographic phase aided in gaining trust of residents and facilitated the recruitment process. A total of 160 qualitative in-depth interviews were conducted between 2015 and 2017 in the various neighborhoods. A maximum diversity sample was employed meaning that adult participants were chosen to include the greatest possible range in terms of age, ethnicity, employment, language, length of residency, religion, and health concerns. This method ensured that a rich range of experiences concerning access to health care was obtained. To facilitate this, participants were recruited with the help of community researchers. Community researchers were residents of the neighborhoods and were employed as part of the project and trained in qualitative methods. The community researchers’ knowledge of the neighborhoods, and their ability to speak various languages, was instrumental in facilitating recruitment, assisting in conducting interviews, and translating during the interviews if the participants did not speak the local language or English (Hamed et al., 2018). Participants were recruited from various local voluntary organizations, migrant associations, and public facilities such as libraries and schools. Snowballing was also used as a way to recruit participants—all while ensuring a maximum diversity of participants. The interviews were conducted in various languages other than Swedish, German, Portuguese, and English depending on the interviewees’ language proficiency and the availability of community researchers.

### Data Selection and Participants

The interviews were conducted with the purpose of examining general barriers to accessing health care, and participant experiences of racism were shared voluntarily. This is important as it highlights the impact of racism on patient experiences and provides space for the participants to shape their own discourse on racism. In this article, we included only interviews (*N* = 11) where an experience of health care was described as having involved or been explicable as a result of discrimination due to ethnicity. The health care experience that was related to discrimination could have been the interviewee’s own or that of a close relative. Interviews where discrimination on the basis of ethnicity was not mentioned, even when health care encounters were negative, were not included in this analysis. All interviewees included here were of migrant background except for one participant, who discussed her mixed ethnicity children’s experience with racism. Some of the participant characteristics are shown in Supplementary Table 1.

### Data Analysis

The data analysis positions participants’ experiences within the wider literature on racism and structural violence that allows the reader to make sense of these experiences within the framing of structural violence. The majority of the interviews were recorded and transcribed using MAXQDA; a data analysis software. The interview guide consisted of questions concerning the participants’ experiences when accessing health care at all levels in connection to their health concerns. Questions concerning barriers, facilitators, and resources used when accessing health care were asked. The interviews were analyzed using deductive thematic analysis, which allowed for a rich exploration of how health care users’ experiences included aspects of structural violence as explained above. Data were coded and analyzed by all authors.

### Ethical Considerations

Ethical approval was obtained from the Ethical Review Committee of the University of Birmingham that is responsible for the study. Ethical approval was also obtained locally in Sweden by the Swedish *etiknämnden* of Uppsala in Sweden as well as the ethical committees of the University of Bremen and the University Institute of Lisbon. All participants in the study received verbal and written information on the aim of the study as well as the way in which their interview would be treated confidentially. Verbal and written informed consent was obtained from all participants. Confidentiality/anonymity and the voluntariness of participation were explained. As the issue of discussing one’s health concern is a sensitive one, participants who were affected by the interview were provided with contact details for a local health care center where they could contact a therapist or psychologist if they so wished. The participants were also given the contact information of the researchers in case they had additional questions or inquiries.

## Findings

In our interviews, health care users reported being deprioritized, treated differently from other patients, as well as being treated with disrespect. This was described by minority health care users who were well educated and integrated in the local society in terms of language proficiency and employment. In our attempt to theoretically frame racism through the lens of structural violence, we identify two processes: (a) unequal access to resources that leads to the silencing of suffering and (b) inequalities in power, which leads to the erosion of dignity.

### Unequal Access to Resources: Silencing of Suffering

I felt that the Swedes get the established doctors while the foreigners get interns . . . it’s a feeling I got . . . but like I said I don’t have proof . . .

This participant, is a Swedish woman of Sudanese origin, points toward the unequal access to resources through health care providers’ decisions to ration care and prioritize treatments, when discussing her perceptions of racism and how minorities are regularly discriminated against. The observation that scarcity of resources often means that it is not possible to manage all health care concerns equally and simultaneously (Scheunemann & White, 2011) does not override the fact that some decisions might be influenced by “race.” However, given that racism is formally unacceptable, it becomes difficult to prove its influence in resource-allocation decisions. A subtle act of rationing and disrespect reported by the health care users in this study can be seen as evidence of the silent workings of structural violence, whose effects are tangible in these experiences.

The conundrum of accessibility to health care resources was described by another participant, a Swedish woman of Somali origin who discussed how her labor pain was not taken seriously, leading to the worst sort of outcome, despite her repeated efforts to get help.

Sometime back I was pregnant and I went to the maternity/delivery hospital because of severe pains and they sent me back home, saying that I was not ready (to give birth). The next day I stopped feeling the baby’s movement and the contractions stopped, so I was worried (. . .) I went to the hospital that evening again, this time I had excruciating pains and they told me that the baby is asleep and you are only 5 centimetres open. They called the doctor and they ran some tests and told me that they think the baby is either dead in my stomach or it is sleeping. So we will check to confirm and then see what to do, either to operate you when the baby is sleeping and/or do a procedure if the baby is dead.

The participant also spoke of other women with Somali background in Sweden who had lost their babies, as they were not offered adequate antenatal care. Even though it is not possible to prove that this rationing of care is a result of “race” as it is often veiled, it works through invisible processes of (non)selection, with tangible effects on individuals. Often minority ethnic women’s expressions of pain in health care encounters are seen as exaggerated and overly emotional (Bowler, 1993) and therefore rationed as a non-priority. We argue that experience such as that described by the above-mentioned participant should not merely be seen as an isolated case but should be situated in the broader context in Sweden where it has been shown that women from Sub-Saharan African countries have statistically significant higher perinatal mortality rate compared with the general population (Essén et al., 2000).

“Big discrimination” were the words used by another participant, a middle-aged health care user of Mozambican background living in Portugal, to explain how he was treated in the emergency room. He received regular health care as he was HIV positive and described how he was ignored at the emergency room after suffering a heart attack even though he tried numerous means of convincing health care providers that his condition was serious.

I waited from 9:30 pm until 2 in the morning to be attended (. . .). They called me around 2 to do the analysis and I complained “I happen to have a heart attack.” “Yes but you will have to wait a little more” and I waited. It was a quarter to three when I was called to do ECO (. . .) “Wow, you are having a heart attack” the guy told me. “No, I have had a heart attack,” I answered. “I am here since 8:30pm (. . .) and I was not attended.

After he was finally attended to and diagnosed, the participant was taken immediately for surgery and stayed at the hospital for more than a week. Here, we note the difference that Galtung (1969) draws between being killed (direct violence) and being allowed to die (structural violence). The veiling of racism conceals the discriminatory processes while the process of rationing through which this occurs is rendered unnoticeable.

However, not all health care encounters that were described as discriminatory led to serious health care outcomes as in the case of the Swedish Somali woman or the Mozambican Portuguese man. Deprioritization rationalized as part of the supposedly neutral and objective medical care is seen in the case of another participant, a 78-year-old German man originally from Sri Lanka. The participant discussed how his wife was discharged without adequate treatment or care at the hospital where she sought care after experiencing an allergic reaction. According to the participant, the nurse treating his wife was “racist” and his wife was not attended despite her continued vomiting and consequent dehydration. As her symptoms continued, his wife sought help again, but avoided going to the previous hospital where she had been ignored.

Okay, then I told the guy (the nurse) I go to the media I’m a reporter. I will tell the press people what is happening in this hospital. Then when she came home and she gave, we gave her this fennel tea. Then she rested early morning again at about 5:00 eh (.) 4:30 or 5:00 within four (.) within six/seven hours again the same problem.

Since this experience, the participant sent letters of complaint to the hospital, to his health insurance provider, his family physician, and the senator for health who heads the Federal Ministry of Health. At the time of the interview, he had received responses from both the hospital and the health insurance provider. The former sent him a complaint form and the latter apparently told him that they abide by what the doctor states, which, in both cases, was experienced as a dismissal of the gravity of his complaint. The dismissal of the participant’s story by the authorities to which he appealed devalued his and his wife’s perception of racism, which became veiled by positioning the health care providers’ narrative as more rational and trustworthy. This resonates with Kirmayer’s discussion on the “partition” of suffering (cited in Farmer, 2004, p. 321)—“the rational sorting of the sick whereby limited resources are concentrated on those who have life threatening but treatable wounds, leaving those with minor wounds to recover on their own and those with moral wounds to die alone.” Even though we have the “capacity for choice,” it is often governed and constrained by “interests that seek to restrict us to a set of options none of which will disturb the system” (Farmer, 2004) and often materializing through the dismissal of experiences of racism as a non-issue as in the case of the Sri Lankan German man and his wife.

A young Swedish man of Chilean background described feeling that as an “immigrant” he did not receive adequate treatment and was not taken seriously and did not receive timely care. Although rationing through the prioritization of more serious illness is a key process in health care, we argue that inequities in health care often masquerade as normal routine medical practices and interactions. The Chilean Swedish man talked about how “White” Swedish patients who, according to him, had no serious illness were prioritized and received treatment while his mother who had a painful migraine attack was left uncared for. His mother was only attended at an emergency clinic when she fainted because of pain. This experience has led this participant to avoid seeking health care as he felt excluded and reduced to an “*invandrare*” or immigrant who is not valued as equal to “White” Swedes. In one case, he told us how he experienced breathing difficulties and chest pain, which he tried to endure without seeking care. When he was finally forced to seek help when his symptoms got worse, he again felt uncared for by the medical staff whom he thought were unwilling to find a cause to his problem. He described how he was the one who suggested the medication, which he found from an internet search, to the doctor, who then prescribed that medication. In an informal conversation with this participant after the interview, he expressed his worry over the growing nationalism in Sweden and a concern that society had become more racist: he was worried, wondering “but what should we do about it?” The growing anti-immigrant sentiments and increasing hate crimes against minorities in Sweden are important, as they constitute part of the participant’s lived experience in health care. The embodied harm experienced by the participant and his mother constitutes a form of structural violence. Structural violence represents “the difference between the potential and the actual, between what could have been and what is” (Galtung, 1969, p. 168).

Much of the experience of perceived discrimination described above was not outright racist expressions by health care providers. Rather withholding of life-saving care was couched within the normal health care routine of rationing. This veiling of racism under rationing makes it difficult, if not impossible, for health care users to express or report their feelings of being deprioritized and neglected. Even when they attempt to report their experiences, as in the case of the Sri Lankan German participant, their experiences are not considered worthy of attention. As already argued, structural violence entails the belittling and silencing of particular groups’ suffering, in this case minority ethnic populations, leading to “unequal life chances, usually caused by great inequality, injustice, discrimination, and exclusion and needlessly limiting people’s physical, social, and psychological well-being” (Uvin, 1998, p. 105). Thus, “individual experience” has to be embedded in the “larger social matrix” to understand how “large-scale social forces (. . .) translate[d] into personal distress and disease” (Farmer, 1996, pp. 261–262).

### Inequalities in Power: Erosion of Dignity

Referring to the “violence continuum” in normative social spaces, Scheper-Hughes (2004) argues that structural violence “refers to the ease with which humans are capable of reducing the socially vulnerable. . . into expendable non-persons” (p. 14). For Farmer, violation of rights is not accidental or random in its distribution or effect. Rather, violations are symptoms of deeper pathologies of power that are linked to social conditions, which determine who will suffer abuse and who will be protected from harm. Farmer (2003) poses the question, “if assaults on dignity are anything but random in distribution or course, whose interests are served by the suggestion that they are haphazard?” (p. 7). In our study, minority health care users felt they were spoken to, treated, and addressed in disempowering ways. These health care users experienced a lack of negotiating power or a lack of participation in the medical decision-making process coupled with a loss of integrity and self-worth. The fact that they perceived themselves to be forgotten and neglected suggests that they had lost a sense of identity (see also Swahnberg et al., 2007). This kind of structural violence is difficult to pin down as it acts mostly through speech where power relations are evident between health care providers and health care users. Thompson (1984) states that “words can be used as instruments of coercion and constraint, as tools of intimidation and abuse, as signs of politeness, condescension and contempt” (p. 42). Variations in accent, vocabulary, and syntax reflect the different positions in the social hierarchy within the medical institution, which gives legitimacy to the words that are spoken.

Farmer (1996, 2003, 2004) argues that structural violence operates in such a way that it renders the marginalized voiceless. Structural violence has the effect of enforcing division as to entitlement to health care but also who is entitled to dignity through patterns embedded in everyday practices of society, including health care. As structural violence is normalized, it is not seen as worth reacting to as demonstrated in the case of another participant, a young German woman of Turkish background who was hospitalized after a car accident. While she was at the hospital, she was greeted by another patient in her room with a Nazi salute.

I had another patient in my room at the hospital; an elderly lady who had broken her arm (. . .). On Saturday morning she started rattling Nazi words [laughs]. Nazi quotes (. . .) My husband got angry and said “Either you change my wife’s room or I complain.” (. . .). At some point the nurse came back in and said I should talk to my husband, he should calm down or I would be driven out now. We then got a new room, I was relocated and the old lady also said goodbye with her arm up (Nazi Salute). But the hospital did not react.

Although the health care providers present in the room saw the Nazi salute being made and although this is an illegal action in Germany, no one intervened, leaving the participant humiliated, sad, and angry that such an act was not reacted to. A plausible explanation could be that such behavior is often excused as psychological imbalance of the performer (the elderly lady). Not only was the case dismissed but it was the participant’s husband who was viewed by health care providers as the troublemaker who needed to calm down. This assault on dignity reproduces violence or in this case violence through racism. Instead of dealing with racism itself, people’s experiences of racism are dismissed and their active objection to racism is constructed as *the problem*. The dismissal of the participant and her husband’s experience of racism veils racism and is in turn veiled within the (re)production of the power relations in medicine where medical staff have power over health care users. This power of the medical personnel and the vulnerability of the woman and her husband, as health care users and as minorities, can be used to dismiss racism and racist encounters in health care as insignificant and a nuisance to the workflow of medical practice.

This Turkish German woman further described how she was treated as a malingerer and a person who faked her illness and pain. This, according to her, led to a delay in diagnosing her disc problem and rheumatism. This is similar to the stereotypes and othering of maternity patients of South Asian descent whose individual behaviors were interpreted by British midwives within stereotypes such as being fussy and lacking maternal instinct (Bowler, 1993).

These relations of power are also reflected in the case of a German woman of Tunisian background who wears the *hijab*. While at the hospital with her ill daughter, the nurses tried to “free” her from her headscarf, which they thought was a symbol of oppression. Instead of focusing on providing care to her daughter who suffers from neurological problems, medical staff who disapproved of the participant’s scarf forced her into a position of defending herself and her religious choices by explaining that she wore the hijab voluntarily. According to her, after she had defended her use of the hijab, nurses treated her with scorn and ignored her. This participant’s story should be read within the general context of reports of growing Islamophobia in Europe resulting from the assumption that Islam is a totalitarian religion that is incompatible with the European (and Western) values of liberty, equality, and democracy (Bayraklı & Hafez, 2016). This perception of Islam as a totalitarian religion incompatible with European values is particularly evident in regard to discussions surrounding the banning of hijab in the name of emancipation (Mondon & Winter, 2017). This form of racism does not necessarily focus on biological heredity but on the superiority of one culture over another. The apparent entitlement that allowed the nurses caring for the German Tunisian woman’s daughter to criticize her choice of headwear can be seen as an example of how racism becomes normalized and hegemonic. Minorities are in these contexts reduced to their migrant identity affecting their negotiating powers in health care. This is reflected in the case of a young German woman originally from Cameroon. When seeking medical care for restricted arm mobility and upon mentioning that she had just come from Africa, the doctor assumed that she could have Ebola. Although she had no symptoms, the doctor refused to examine her and instead proceeded to google whether there was Ebola in Cameroon and, according to the Cameroonian German woman, looked scared. Women patients can be infantilized and reduced to the single identity of “migrant” (Lindqvist & Wettergren, 2017), which in this woman’s case was the orientalist constructed category of the “sick African” from the “dark continent.” In various ways and in different locations, then, structures of inequality continue which limit the social power, choices, and life chances of subjects, resonating with Farmer’s (1996) argument on the “exoticization” of suffering, whereby, “the suffering of individuals whose lives and struggles recall our own tends to move us; . . . [While] those who are distanced, whether by geography, gender, ‘race,’ or culture, is sometimes less affecting” (Farmer, 1996, p. 272).

A Swedish Somali woman, in her mid-50s, who suffered from multiple illnesses including heart problems, fibromyalgia, and meningitis, talked about difficulties accessing health care. She had language difficulties although she had lived in Sweden for 7 years. Due to her illnesses and difficulties in mobility, her daughter often assisted her. Her daughter, who worked as an interpreter, was fluent in Swedish and was present during the interview. In general, the participant and her daughter reported how they experienced difficulties in receiving adequate treatment especially considering the participant’s complex health issues. Although, they did not explicitly attribute all their difficulties to discrimination, the participant stated that even though she was treated well by many nurses, she felt discriminated against and treated rudely by some of the nurses at the hospital. In one case, they explained how one nurse refused to do the Internationalized Normalized Ratio (INR) test that was needed regularly to monitor how long it took for the woman’s blood to clot since she was on an anticoagulant medication. According to the participant, the test should be taken without an appointment as per her doctor’s instructions, but nurses had taken up a gatekeeping role and she felt she had to negotiate and argue for a test that she had the right to.

It’s (the INR test) is on drop in: you go to the hospital and do the test. But sometimes when we (the participant and her daughter) talked to the nurses, I felt some of them were really nasty and no we didn’t need a referral but they (the nurses) kept asking for it (. . .) they were very rude.

Another health care user, a White Swedish woman, said that a health care provider referred to her son, who is both White Swedish and Eritrean, as exotic. Although the woman did not have a foreign background herself, she described how her children experienced racism in health care as well as in other settings. As she works herself in the health care system, she was able to access her son’s patient notes where she found the description “exotic” attributed to her son. She also explained that the same health care provider that had used the term “exotic” thought that she and her daughter were lying about a mole. The health care provider was not convinced that the mole was real because he considered it too dark and accused the participant and her daughter of drawing it with a pen. The mole was later surgically removed.

When the same doctor met my daughter (. . .) she had a small mole (. . .) he thought that she had a very dark mole so he asked if she had drawn it with pen . . . because for him it was too dark.

The participant connected what happened in health care to other incidents where her children were targeted because of their skin color. When telling these stories, the participant seemed very emotional and upset about what happened, stating, “sometimes you are with your Swedish friends and they are very nice and friendly and then suddenly they start talking like a Swedish Democrat” referring to the anti-immigrant nationalist political party in Sweden.

An older Indian Indonesian man, who had lived in Sweden for more than 30 years, has been deeply affected by incidents of disrespect in his health care encounters. He was generally discontent with health care providers in Sweden whom he described as “useless” as they render him and his health concerns invisible. He discussed how he was mocked by a health care provider at the hospital, because of his Indian background. He had lost one of his eye lenses in the late 1980s after a failed cataract operation in Sweden. He explained that he tried to contact some Indian surgeons in India, to get some consultation on how to replace his lens. The reasons why he contacted doctors outside Sweden was his inability to access suitable help in Sweden, as well as his general mistrust of the system. When he was finally able to get an appointment with a doctor in Sweden, he described to the Swedish doctor his attempts trying to contact doctors in India but was mocked. The doctor stated sarcastically, “I am certain that your Indians most probably can repair it,” meaning that Indian doctors may claim to treat anything, but implying that they were nonetheless incompetent. The participant also mentions another incident at a primary health clinic where a doctor treated him and his wife harshly and mistook his missing lens as some kind of “deadly infection.” Structural violence has left the participant feeling not only humiliated but also forced to seek health care in other countries instead.

Being overlooked was also reported by a Sudanese Swedish woman who talked about how she was disregarded by a nurse when she was admitted to hospital for surgery.

I thought that my nurse I don’t know what to call her, a native or what I should call her; I felt that the way she treated me was not good . . . I didn’t like it . . . some of the things that she could have done in the room, she would take me somewhere else without explaining why . . . sometimes I asked her if I could eat now and she would tell me “No, you can’t! This is not the time to eat.” She treated me in a tough way, she wasn’t nice at all. I actually felt sorry for her more than sorry for myself.

The vagueness of the complaint that the above participant makes illustrates the indirect operation of this abuse, which precludes it from being recognized as racism. However, these stories of pain, suffering, and indignity have a negative effect on patients’ willingness to consult health care providers and may have an impact on health status. Despite these experiences, none of those interviewed tried to issue a formal complaint or discuss this issue with the health care center in which the incidents happened. We have argued elsewhere that in a context of anti-immigrant national politics, people of migrant background and particularly women from outside Europe feel an obligation to be “good” migrants, who express gratitude for health services and not be overly demanding (Bradby et al., 2018). This is because of the insidious nature of structural violence, which acts implicitly making it difficult to prove as illustrated by the Sudanese Swedish women’s quote above, describing her sense that her care was consistently deprioritized as “a feeling I got . . . but like I said I don’t have proof.”

The normalization of structural violence makes racial discrimination readily dismissed as a non-issue. Instead of trying to face the issue, health care users either resorted to changing health care centers and providers or used strategies such as spending time to convince health care providers that their symptoms were real which in some cases resulted in a loss of trust and avoidance of health care services. This may lead to increased suffering as not only are integrities lost and symptoms deemed insignificant but also the responsibility of negotiating with the health care system becomes that of the patient rather than a professional responsibility or a structural issue.

## Concluding Discussion

This article has explored the accounts of 11 health care users in three European countries on their experiences of racism in health care encounters through the lens of structural violence. These interviews are embedded in the political context of “European Exceptionalism”—an increasing anti-immigrant nationalist discourse and a sense that minority populations are less deserving of health care services. This moral high ground brushes aside experiences of racism as unreal or exaggerated. In this article, we analyze how racism as an institutionalized social structure is enacted through subtle, invisible practices that are normalized into everyday medical routines, which eventually limit or deny access to basic human needs and a humane quality of life. It can make particular subjects vulnerable, unable to protect themselves, and produce barriers for the individual’s ability to seek specific help in response to this.

Our article situates these individual experiences within the “violence” inherent to the social structure of racism and racial inequalities. As the health care users in this study have indicated, the ability of the medical staff to view a patient’s narratives as illegitimate, unworthy of treatment or delay in treatment, can be influenced by racial stereotypes. These stereotypes thus affect the diagnostic process, which in itself is a site of negotiation between health care users and health care providers.

Racism is a violation of the health care professions’ ethics that seek to provide care equitably as a form of social solidarity. For medical professions that hold the ideal of avoiding maleficence toward the patient as a core ethical principle, racism represents a particular challenge to the professional identity, which may explain some of the difficulty in discussing racism in the public discourse. Health care providers have high levels of autonomy and strong professional identities that are often based on an idea of scientific rationality, which further complicates the discussion of racism. The need to defend rationality supports a dismissal of racism within health care, which is also connected to the dismissal of racism as an important historical process in the formation of European modernity. To facilitate discussion and promote the eradication of the damaging effects of racism in health care settings, there is a need to develop an understanding of why racism is so problematic to identify in public discourse. This analysis using ideas of structural violence shows one way to illuminate the mechanisms of subtle racism that damage individuals and undo trust in health care providers. This analysis can be used as an effort to create constructive public discussion with health care providers and users.

### Limitations of the Study

The interviews were part of a larger project that did not focus on racism but rather on general barriers to health care. Exploration of minorities’ experiences of racism in health care may be lacking, as interviewers may have not gone in depth into these experiences. We do not claim that structural violence is the only form of violence that can adequately explain the complexity of structural forces and individual actions within health care settings. Thus, we are aware that structural violence interfaces with other forms of violence, though, in this article, we focus on structural violence while remaining aware that other violences may exist alongside it.

## Supplemental Material

sj-pdf-1-qhr-10.1177_1049732320931430 – Supplemental material for Racism in European Health Care: Structural Violence and Beyond Click here for additional data file. Supplemental material, sj-pdf-1-qhr-10.1177_1049732320931430 for Racism in European Health Care: Structural Violence and Beyond by Sarah Hamed, Suruchi Thapar-Björkert, Hannah Bradby and Beth Maina Ahlberg in Qualitative Health Research
