# Supplementary material for: Racism in European Health Care: Structural Violence and Beyond
Source: Qual Health Res. 2020 Jun 16;30(11):1662–73. doi: 10.1177/1049732320931430 (PMC7410275; doi:10.1177/1049732320931430)
Supplement: sj-pdf-1-qhr-10.1177_1049732320931430 – Supplemental material for Racism in European Health Care: Structural Violence and Beyond [file sj-pdf-1-qhr-10.1177_1049732320931430.pdf]

Supplementary Material

Table S1: Participant characteristics (N=11)

	N (%)
Gender	
Male	4 (36%)
Female	7 (64%)
Age[a], Mean	47.7
20-30 years	1 (9%)
30-40 years	2 (18%)
40-50 years	4 (36%)
Over 50 years	4 (36%)
Marital status	
Married	7 (64%)
Single	2 (18%)
Divorced	1 (9%)
Widow	1 (9%)
Employment	
Employed	6 (55%)
Non employed	2 (18%)

Student	1 (9%)
Pensioner	2 (18%)
Unspecified	1 (9%)
Religion	
Islam	5 (45%)
Christianity	2 (18%)
Hindu	1 (9%)
Non practising Sikh	1 (9%)
Atheist/Agnostic	2 (18%)
Ethnicity	
African	1 (9%)
Somali	1 (9%)
Chilean	1 (9%)
Turkish	1 (9%)
Indian	1 (9%)
Not specified	4 (36%)
Time living in neighbourhood	
5-10 years	2 (18%)
10-20 years	3 (27%)

20-30 years	1 (9%)
Over 30 years	5 (45%)
Language proficiency	
Fluent	8 (72%)
Non-Fluent	3 (27%)

[a] at the time of the interview
[b] as identified by the participant
